# The evolutionary conservation of the core components necessary for the extrinsic apoptotic signaling pathway, in Medaka fish

**DOI:** 10.1186/1471-2164-8-141

**Published:** 2007-06-01

**Authors:** Kazuhiro Sakamaki, Masami Nozaki, Katsuya Kominami, Yutaka Satou

**Affiliations:** 1 Department of Animal Development and Physiology, Graduate School of Biostudies, Kyoto University, Kyoto 606-8501, Japan; 2 Department of Cell Biology, Research Institute for Microbial Diseases, Osaka University, Suita 565-0871, Japan; 3 Department of Zoology, Graduate School of Science, Kyoto University, Kyoto 606-8502, Japan; 4 Present address: Nihon Schering Research Center, Kobe 650-0047, Japan

## Abstract

**Background:**

Death receptors on the cell surface and the interacting cytosolic molecules, adaptors and initiator caspases, are essential as core components of the extrinsic apoptotic signaling pathway. While the apoptotic machinery governing the extrinsic signaling pathway is well characterized in mammals, it is not fully understood in fish.

**Results:**

We identified and characterized orthologs of mammalian Fas, FADD and caspase-8 that correspond to the death receptor, adaptor and initiator caspase, from the Medaka fish (*Oryzias latipes*). Medaka Fas, caspase-8 and FADD exhibited protein structures similar to that of their mammalian counterparts, containing a death domain (DD), a death effector domain (DED) or both. Functional analyses indicated that these molecules possess killing activity in mammalian cell lines upon overexpression or following activation by apoptotic stimuli, suggesting similar pro-apoptotic functions in the extrinsic pathway as those in mammals. Genomic sequence analysis revealed that the Medaka *fas *(*tnfrsf6*), *fadd *and *caspase-8 *(*casp8*) genes are organized in a similar genomic structure as the mammalian genes. Database search and phylogenetic analysis revealed that the *fas *gene, but not the *fadd *and *casp8 *genes, appear to be present only in vertebrates.

**Conclusion:**

Our results indicate that the core components necessary for the extrinsic apoptotic pathway are evolutionarily conserved in function and structure across vertebrate species. Based on these results, we presume the mechanism of apoptosis induction via death receptors was evolutionarily established during the appearance of vertebrates.

## Background

Apoptosis, a major form of cell death, is a significant biological phenomenon that removes unnecessary, superfluous, damaged or harmful cells in multicellular organisms. Apoptosis is important for tissue morphogenesis during development, maintenance of homeostasis in adulthood, and defense and immune responses [1-5]. In apoptosis, activation of a family of cysteine proteases known as caspases induces the proteolytic cleavage of many critical proteins, leading to cell suicide [6]. In mammals, 15 caspases have been identified. Of these, caspases-2, -8, -9 and -10 play roles as initiators, while caspases-3, -6 and -7 function as downstream effectors. The activation of effector caspases is the converging point of two major signal pathways: the extrinsic pathway initiated by ligation of cell surface receptors called "death receptors", including Fas (APO-1/CD95) and receptors for tumor necrosis factor-related apoptosis-inducing ligand (TRAIL), and the intrinsic pathway triggered by cytochrome c release from mitochondria into the cytosol.

The extrinsic apoptotic signaling pathway following Fas ligation has been well characterized [7,8]. Oligomerization of Fas by its natural ligand or an agonistic antibody recruits the adaptor molecule FADD (Fas-associated death domain protein, also termed MORT1) [9,10] to the death domain (DD) of the Fas intracellular region. Procaspase-8 (also known as FLICE/MACH1/Mch5), which is an inactive zymogen, associates in turn with FADD by interactions between their death effector domains (DED) [11,12]. Within the Fas-FADD-procaspase-8 complex, called the death-inducing signaling complex (DISC) [13], procaspase-8 undergoes auto-cleavage to convert to an active form. Through cleavage, activated caspase-8 activates downstream effector caspases and Bid, a member of the Bcl-2 family, eventually leading to cell death [14-16]. Deficiency in caspase-8 leads to suppression of Fas-mediated apoptosis [17-19].

Although most studies examining the extrinsic apoptosis pathway have utilized mammalian systems, homologs of the apoptosis signaling molecules, including death receptors and caspases, have recently been identified in zebrafish (*Danio rerio*), including two death receptors, the zebrafish hematopoietic death receptor (ZH-DR) and the ovarian TNFR (OTR) [20,21]. Caspase-3 and two additional caspases that are homologous to human caspases-1 and -5 have been characterized in zebrafish [22,23]. Caspases-3, -6, -7 and -9 are also identified and characterized in salmon and sea bass [24,25]. Several genes with homology to mammalian regulators of apoptosis, including *caspase-8*, *bid *and *fadd*, have been identified in the zebrafish [26-28]. Thus, the apoptotic machinery appears to be conserved between fish and mammals. No extensive functional analyses of these apoptotic regulators have been performed in fish.

To understand the general mechanisms regulating cell death in vertebrates, we studied the apoptotic machinery governing the extrinsic signaling pathway in fish. In this study, we identified and characterized orthologs of mammalian Fas, FADD and caspase-8 that might be indispensable for extrinsic apoptotic signaling in Medaka fish (*Oryzias latipes*). We report that these molecules act as pro-apoptotic molecules and are able to substitute for the functions of their mammalian counterparts in mammalian cells. These results suggest the evolutionary conservation between fish and mammals of the core components essential for the extrinsic pathway. We also discuss the development of the extrinsic apoptotic signaling pathway in conjunction with the appearance of vertebrates during evolution.

## Results

### Primary structure of Medaka Fas, FADD, and Casp8 molecules

**Figure 1:**
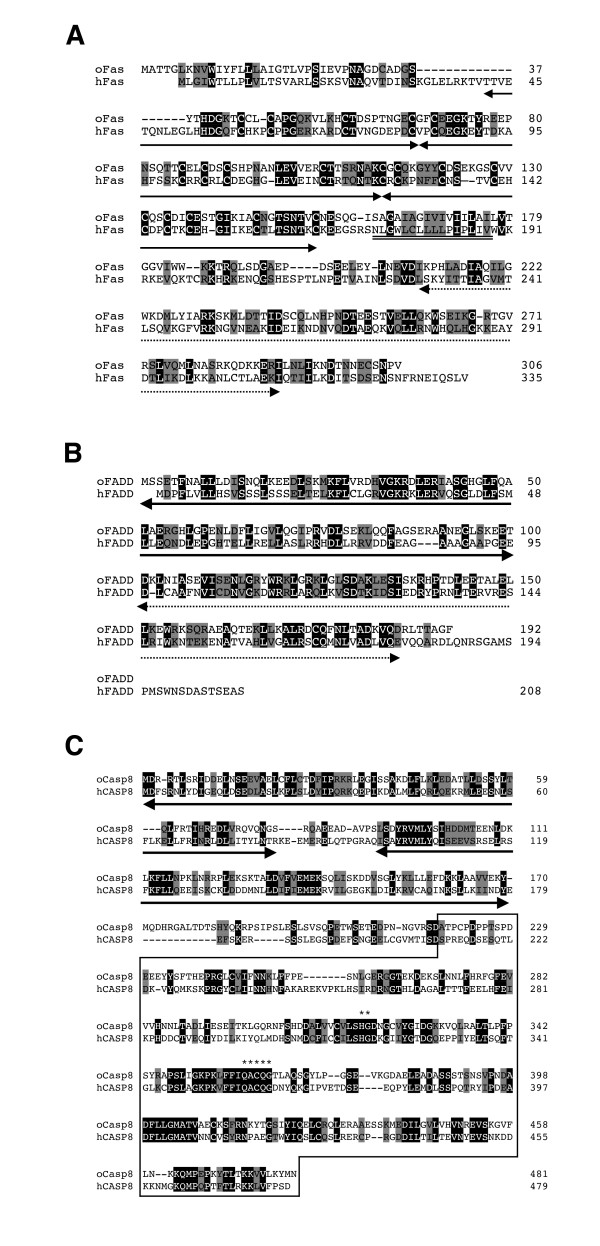
**Amino acid sequence comparison of Fas, FADD and caspase-8 between Medaka and humans**. (A) Alignment of Medaka and human Fas. The three lines underneath the sequence indicate the cysteine repeat domains in the extracellular region. The double line and the dotted line indicate the transmembrane region and the death domain (DD) within the cytoplasmic region, respectively. (B) Alignment of Medaka and human FADD. The bold and dotted lines under the sequence indicate the death effector domain (DED) and the DD, respectively. (C) Alignment of Medaka and human caspase-8 (CASP8). The two bold lines and a box indicate the DEDs and the protease domain, respectively. Asterisks represent the amino acids essential for catalytic activity. Identical and similar amino acids between Medaka and human in all alignments are indicated in black and shaded boxes, respectively.

We searched the GenBank DNA database for the fish homologs of mammalian *FAS *(*TNFRSF6*), *FADD *and *caspase-8 *(*CASP8*), three essential components of Fas-mediated apoptotic signaling. We identified an expressed sequence tag (EST) clone ([GenBank: AU176749]) similar to *FAS*, an EST clone ([GenBank: AU242372]) similar to *FADD *and two EST clones ([GenBank: BJ006125] and [GenBank: AV670945]) similar to *CASP8 *in the Medaka cDNA library. Sequencing of these EST clones confirmed that the full-length cDNAs encoded open reading frames of 306, 192, and 481 amino acids, exhibiting homology to Fas, FADD and caspase-8, respectively, by a BLAST search [29]. Protein structure analysis using the Pfam database [30] showed that the predicted Fas-like molecule contained a DD in the cytoplasmic region, the FADD-like molecule possessed both DED and DD, while the caspase-8-like molecule contained two DEDs and a protease domain. Therefore, these proteins might be orthologs to mammalian Fas, FADD and caspase-8; we termed the molecules encoded by these cDNA clones Medaka Fas, FADD and caspase-8. Alignments of the Medaka and human Fas, FADD and caspase-8 molecules revealed 24%, 34% and 34% identity and 42%, 52% and 52% similarity at the amino acid sequence level, respectively (Figure 1). Comparison of Medaka Fas to the zebrafish death receptors, zebrafish hematopoietic death receptor (ZH-DR) and ovarian tumor necrosis factor receptor (OTR) [20,21] identified 22% and 20% identity at the amino acid level, respectively. These results suggest that Medaka Fas is more similar to human Fas than to fish ZH-DR and OTR. Thus, we identified structurally similar molecules potentially orthologous to mammalian Fas, FADD and caspase-8, core components of the extrinsic apoptotic signaling pathway, in the Medaka fish.

### Genomic structure of the Medaka *fas*, *fadd *and *casp8 *genes

**Figure 2:**
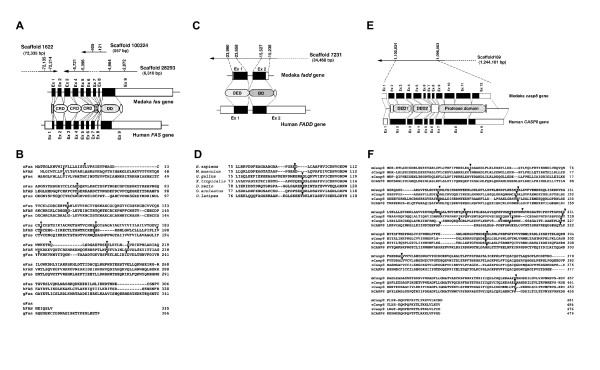
**Analyses of the genomic structure of the Medaka *fas, fadd *and *casp8 *genes**. (A) Genomic organization of the Medaka and human *FAS *(*TNFRSF6*) genes. Both Medaka and human *FAS *are composed of nine exons. The coding and non-coding regions are indicated by black and white boxes, respectively. The majority of the exons of the Medaka *fas *gene were identified in the genomic sequence from scaffold1622 (exon 1: 72, 135-72, 214), scaffold28293 (exons 3 and 4: 5, 721-5, 396; exons 7–9: 4, 964-2, 872) and scaffold100324 (exons 5 and 6: 405-121). (B) Comparison of the splice junction sites in Medaka, human and chicken Fas. Arrowheads on amino acid alignment indicate the splice junction sites in the Medaka, human and chicken genes, respectively. (C) Genomic organization of the Medaka and human *FADD *genes. Both the Medaka *fadd *and human *FADD *genes contain two exons. The black and white boxes correspond to the coding and non-coding regions, respectively. The *fadd *gene was detected from the genomic sequence (23, 960-15, 238) of scaffold7231. (D) Comparison of the splice junction sites of FADD from several vertebrates. An arrowhead indicates the splice junction site, defined by the comparison of the cDNA and the genomic sequences of human (*H. sapiens*), mouse (*M. musculus*), chicken(*G. gallus*), frog (*X. tropicalis*), zebrafish (*D. rerio*), stickleback (*G. aculeatus*) and Medaka (*O. latipes*). (E, F) Genomic organization of the Medaka and human *CASP8 *genes. The Medaka *casp8 *gene was identified in the genomic sequence (1, 102, 834-1, 096, 083) of scaffold169 (E). The Medaka *casp8 *gene is composed of 12 exons, while the human *CASP8 *gene is 9 exons. The coding and non-coding regions are indicated by black and white boxes, respectively. Comparison of the splice junction sites in Medaka, stickleback, zebrafish and human caspase-8 is shown in (F). Arrowheads on amino acid alignment indicate the splice junction sites in the Medaka, stickleback, zebrafish and human genes, respectively. Abbreviations: CRD, cysteine repeat domain; DD, death domain; DED, death effector domain; TM, transmembrane domain.

To confirm that the isolated Medaka Fas, FADD and caspase-8 are orthologs of their mammalian counterparts, we examined the genomic structure of the *fas *(*tnfrsf6*), *fadd *and *caspase-8 *(*casp8*) orthologous genes in fish, mammals and birds. By comparing the genomic and the cDNA sequences, we determined the organization of the Medaka *fas*, *fadd *and *casp8 *genes. The Medaka *fas *gene consisted of 9 exons and 8 introns, corresponding to those in the human and chicken *FAS *gene with coincident splice junction sites (Figures 2A, 2B). The nucleotide sequences of the exon-intron boundaries in the Medaka *fas *gene [see Additional file 1] completely conformed to the GT-AG rule [31]. The Medaka *fadd *gene possessed two exons, identical to that of the human gene (Figure 2C). Additionally, search of the GenBank and Ensembl genome databases revealed that the *fadd *genes in the human (*Homo sapiens*), mouse (*Mus musculus*), chicken (*Gallus gallus*), West African clawed frog (*Xenopus tropicalis*) and two species of fish, stickleback (*Gasterosteus aculeatus*) and zebrafish (*Danio rerio*) consisted of two exons (Figure 2D). In the Medaka *fadd *gene, the GT-AG rule was also observed at the exon-intron boundaries [see Additional file 2]. By comparing genomic sequences to the obtained Medaka *casp8 *cDNA sequence, we confirmed that the *casp8 *gene consists of 12 exons and 11 introns (Figures 2E, 2F). The nucleotide sequences of the exon-intron boundaries of the Medaka *casp8 *gene agree with the GT-AG rule [see Additional file 3]. The genomic organization of the Medaka *casp8 *gene was similar to that of the human and chicken *CASP8 *genes with mostly coincident splice junction sites, but additional exons comprising a portion of the first DED and the protease domain were present in the Medaka *casp8 *gene (Figure 2F). We also determined the genomic structure of the stickleback and zebrafish *casp8 *genes, and detected additional exons in the stickleback *casp8 *gene but not the zebrafish *casp8 *gene (Figure 2F). This corresponded to the phylogenetic relationships, indicating that Medaka and stickleback are more closely related to each other than either of them is to zebrafish [32]. Thus, these conserved structures of the *Fas*, *FADD *and *Casp8 *genes within vertebrates strongly suggest that these genes share their origin respectively.

### Genomic organization of the Medaka *fas*, *fadd *and *casp8 *genes

**Figure 3:**
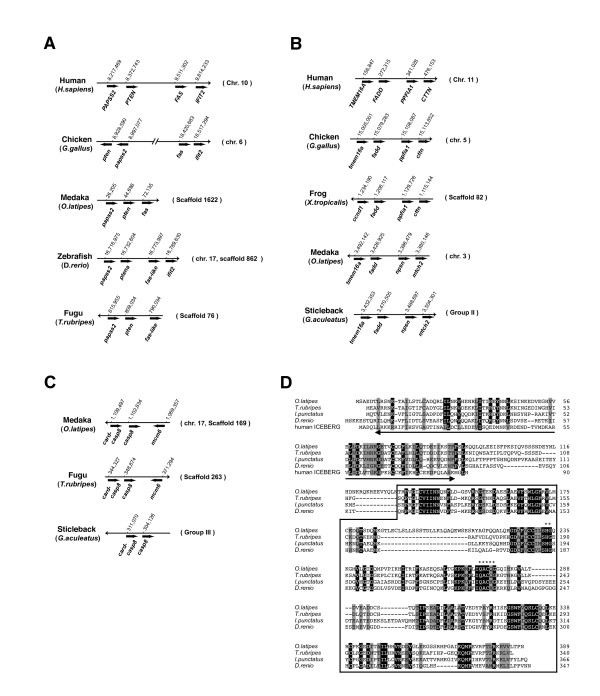
**Chromosomal analyses of the Medaka *fas, fadd *and *casp8 *genes and their orthologs**. (A) A physical map of the region containing the *FAS *gene was generated for human, chicken and fish. In human and chicken, the *FAS *gene localizes to a locus on Chr. 10q24.1 or chr. 6. In Medaka, the *fas *gene resides close to the *pten *gene in the genome. In the zebrafish genome, the *fas-like *gene is located between the *ptena *and *ifit2 *genes. The fugu *fas-like *gene is also found near the *pten *gene. (B) A physical map of the region containing the *FADD *gene was generated for human, chicken, frog and fish. In human and chicken, the *FADD *gene localizes to a locus on Chr. 11 or chr. 5. The Medaka *fadd *gene is located between the *tmem16a *and *npsn *genes on chr. 3. In stickleback, the region containing the *fadd *gene shows a similar synteny. (C) A physical map of the region containing the *casp8 *gene was generated for Medaka, fugu and stickleback. The *casp8 *gene localizes between the *card-casp8 *and *mcm6 *genes in both Medaka and fugu genomes. In the stickleback genome, the *casp8 *and *card-casp8 *genes also localize in tandem. (D) Multialignment of the Medaka Casp8-like molecule (CARD-Casp8) and its homologs identified in fish. The *card-casp8 *genes from fugu, catfish ([GenBank: AY555576]) and zebrafish were identified by database search. Human ICEBERG ([GenBank: P57730]), which is a CARD-only molecule, was cited for reference. Identical and similar amino acids in all alignments are indicated in black and shaded boxes, respectively. The underline and a box indicate the CARD motif and the protease domain, respectively. Asterisks represent the amino acids essential for catalytic activity.

To understand the evolutionary divergence or conservation of the core molecular components required for the extrinsic apoptotic signaling pathway, we next examined the genomic location of the *fas *gene in vertebrates by computational analysis. The human *FAS *gene localizes to chromosome 10 (Chr. 10), according to LocusLink (Figure 3A). Search of the zebrafish genome sequence in the Ensembl database identified a *fas-like *gene on scaffold862 that was highly homologous to the human and Medaka *FAS *genes [see Additional file 4]. Within the assembly, this scaffold corresponds to chr. 17, and we found a syntenic relationship between this zebrafish genomic region and the human chromosomal segment including the human *FAS *gene. Putative orthologs of *PAPSS2*, *PTEN *and *IFIT2*, which co-localize with the *FAS *gene on human Chr. 10 are found in close proximity to the *fas-like *gene in the zebrafish genome. This gene organization was also conserved in the chicken chr. 6 and at least partially in the Medaka and fugu (*Takifugu rubripes*) genomes (Figure 3A). In addition, comparative mapping analysis predicted the presence of the *fas *gene in the frog genome [see Additional files 5, 6]. Therefore, the genomic organization around the *Fas *gene locus exhibits synteny throughout vertebrates.

Genomic organizations around the *FADD *gene were also well conserved among the vertebrate genomes (Figure 3B). In the human genome, the *CCND1, ORAOV1, FGF19, FGF4, FGF3, TMEM16A, FADD, PPFIA1 *and *CTTN *genes are encoded in this order on Chr. 11. This gene order is also conserved in the chicken genome. In the fish genomes, the gene order from *ccnd1 *to *fadd *is also conserved [33], although the *ppfia1 *and *cttn *genes, which are 3' neighbors of the *fadd *gene, were replaced with *npsn *and *mtch2*. The only exception we found was the *X. tropicalis *genome. In this genome, the chromosomal segment from *oraov1 *to *tmem16a *is translocated and thus the gene order in this genome is *ccnd1-fadd-ppfia1-cttn*. This data suggests the overall syntenic conservation of the genomic regions around the *fadd *gene within the vertebrates and that a chromosomal rearrangement event in a genomic region downstream of the *fadd *gene has occurred after the divergence of fish and other vertebrates (the rearranged organizations are also conserved in each lineage).

We investigated the genomic organization of the *casp8 *gene in fish and other vertebrates, and the chromosomal segment around this gene is conserved between fish and other vertebrates [34]. In the Medaka genome, a putative ortholog of the *mcm6 *gene, which localizes close to the *CASP8 *gene on human Chr. 6, resides near the *casp8 *gene (Figure 3C). The orientation and position of *casp8 *and *mcm6 *genes were consistent with those identified in the fugu genome database. Moreover, upstream of the *casp8 *gene, a *casp8-like *gene was identified with a region encoding the protease domain possessing the caspase specific active site QACQG (Figure 3D). The predicted amino acid sequence of the Casp8-like molecule was highly similar to the protease domain of mammalian caspase-8 proteins (Casp8) by BLASTP searches. Phylogenetic studies also suggested that the protease domain of the Casp8-like molecule is most similar to that of CASP8 but not to that of other caspases [34]. The putative orthologous genes for this Medaka *casp8-like *gene were also found in the fugu, stickleback and zebrafish genomes, localizing next to the *casp8 *gene (Figure 3C and [34]). These gene products similar to Casp8 lack two DED motifs in its N-terminus but contain a caspase recruitment domain (CARD), which were revealed by the InterProScan program using the Interpro database (Figure 3D). In the present study, this *casp8-like *gene was tentatively termed as *card-casp8*. Therefore, we suggest that the *card-casp8 *gene is derived from a copy occurred by a tandem duplication of the ancestral *casp8 *gene in the fish lineage, and that one of the duplicated copies might acquire a CARD and probably a new function at the same time.

### Molecular phylogenetic analysis of Fas, FADD and Casp8

**Figure 4:**
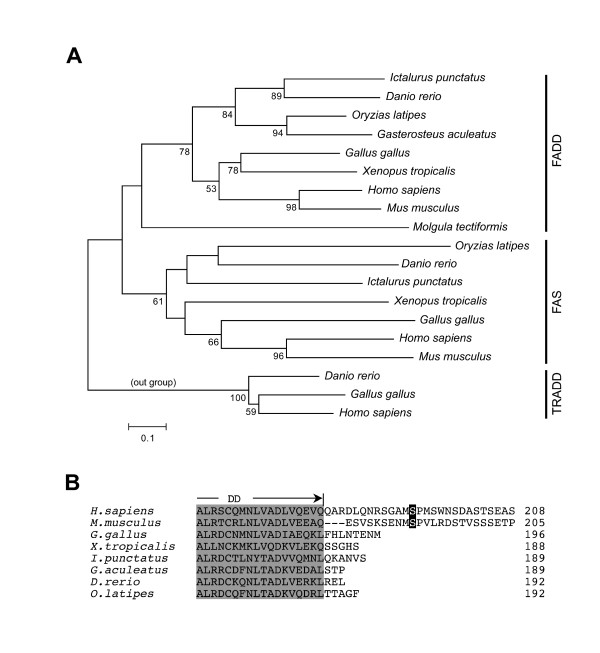
**Molecular phylogenetic analysis of Fas and FADD molecules**. (A) A molecular phylogenetic tree of Fas and FADD generated by the neighbor-joining method based on an alignment of death domains. Human, chicken and zebrafish TRADD were used as the outgroup proteins for rooting the tree. The number noted at branches indicates the percentage of times that a node was supported in 1000 bootstrap pseudoreplications and are shown only greater than 50% for the bootstrap value. The scale bar indicates an evolutionary distance of 0.1 amino acid substitutions per position. All of known or identified Fas and FADD proteins in ascidian (*Molgula tectiformis*), catfish (*Ictalurus punctatus*), chicken(*Gallus gallus*), human (*Homo sapiens*), Medaka (*Oryzias latipes*), mouse (*Mus musculus*), stickleback (*Gasterosteus aculeatus*), West African clawed frog (*Xenopus tropicalis*) and zebrafish (*Danio rerio*) were included in this tree. (B) Multialignment of the C-terminus of FADD. Amino acids in a DD are indicated in shaded boxes. A phosphorylated serine residue in human and mouse FADD is indicated by a black box. Analyzed animals: human, mouse, chicken, frog, catfish, stickleback, zebrafish and Medaka.

Domain organizations, genomic structures and syntenies between the vertebrate genomes strongly suggest that these Medaka genes identified in this study are true orthologs for *Fas*, *FADD *and *Casp8*. Molecular phylogenetic analyses for these genes also reinforced the orthologies of these genes. For constructing a molecular phylogenetic tree for FAS and FADD proteins, we used Fas and FADD sequences manually predicted from the chicken, zebrafish and stickleback genomes together with published catfish (*Ictalurus punctatus*), frog, human and mouse Fas and FADD sequences [9,10,35-40] [see Additional file 7]. A molecular phylogenetic tree for the Fas and FADD families based on an alignment of the death domain indicated that Medaka Fas and FADD were most similar to fish Fas and FADD, respectively (Figure 4A). The generated tree suggests that Medaka Fas is closest to zebrafish Fas-like molecule (bootstrap value = 44%), and supports its orthology for Fas proteins encoded in the genome of amphibians and amniotes (bootstrap value = 61%). Additionally, we could not find any typical death receptors bearing a DD in invertebrates from the public databases (data not shown). This was supported by reports describing no orthologs of the death receptors in spite of extensive screens of decoded genomes, even in the genome of *Ciona intestinalis*, a close relative of vertebrates [41,42]. Therefore, these lines of evidence suggest that the death receptor such as Fas appears early during vertebrate evolution.

Similarly, the molecular phylogenetic tree suggested that fish FADD proteins including Medaka FADD are monophyletic (bootstrap value = 84%), and these proteins are orthologous for FADD proteins encoded in the genome of amphibians and amniotes (bootstrap value = 78%). However, alignment of the C-terminal amino acid sequences of these FADD molecules revealed diversity among mammals and other vertebrates including fish (Figure 4B). In the C-terminal portion of the molecule neighboring the DD, human and mouse FADD are phosphorylated at Ser residues (194 in human and 191 in mouse) for the regulation of mitosis [43,44]. This C-terminal extension is not present in birds, frogs, or fish. A gene encoding a putative FADD is also found in an ascidia, *Molgula tectiformis*. Although the *Molgula *FADD molecule certainly possesses both DED and DD motifs, it is highly divergent from that of vertebrates (Figure 4A). Moreover, a gene for FADD was identified in *Drosophila*, but the structure of *Drosophila *FADD is distinct from those of chordate FADD molecules because of lacking a definite DED [45]. Therefore, the origin of FADD, which might be present in a common ancestor of chordates, is older than that of Fas, which may have arisen in the vertebrate lineage. Similarly, Medaka *casp8 *is suggested to be a member of the caspase-8 and caspase-10 family, (caspase-10 is a putative paralogous gene of caspase-8). Thus, the genes encoding core components of the extrinsic signaling pathway, Fas, FADD and Casp8, are present in the fish genome but are not completely in the non-vertebrate chordate and other invertebrate genomes.

### Pro-apoptotic activity of Medaka Fas, FADD and Casp8

**Figure 5:**
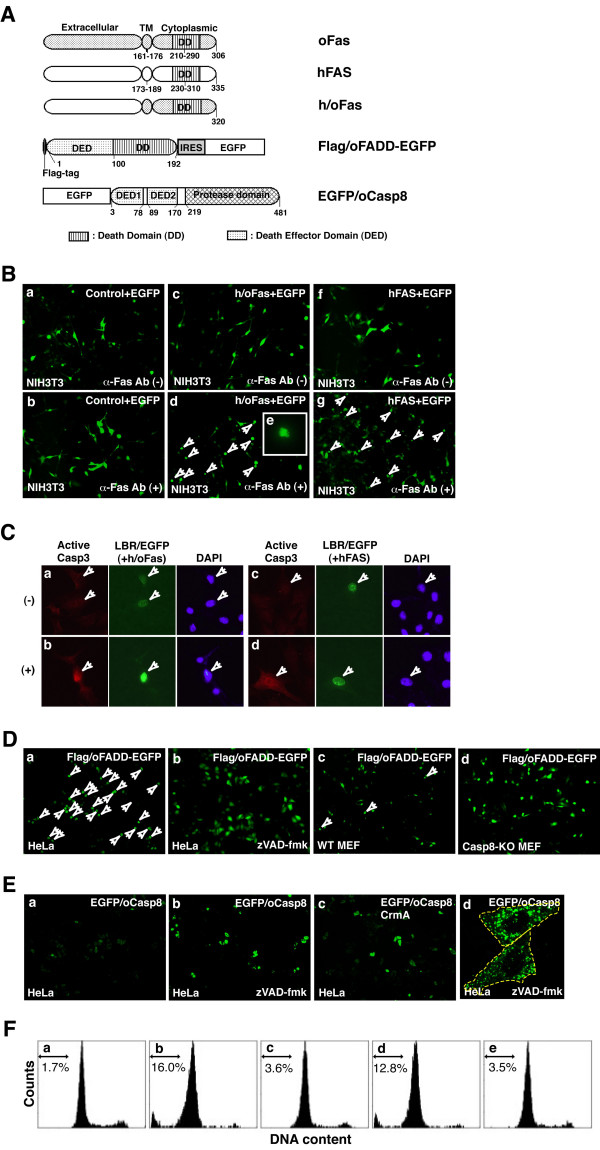
**Functional analyses of Medaka Fas, FADD and Casp8**. (A) Schematic diagram of the plasmid constructs for the expression of human and Medaka chimeric Fas (h/oFas), Flag/oFADD-EGFP and EGFP/oCasp8 proteins. The chimeric h/oFas consists of the extracellular domain of human FAS and the transmembrane and cytoplasmic regions of Medaka Fas. The Flag/oFADD-EGFP construct translates both Flag-tagged Medaka FADD and EGFP molecules from a bicistronic mRNA. The EGFP/oCasp8 is a fusion of Medaka caspase-8 with EGFP at the N-terminus. (B) Cytotoxicity assays of chimeric Fas introduced into mouse NIH3T3 cells. Empty pME18S vector (panels a and b), pME18S-h/oFas (panels c, d and e) or pME18S-hFAS (panels f and g) was cotransfected transiently with pEGFP-C1 into NIH3T3 cells. After culture for 48 h, these transfectants were incubated for 14 h in the presence (panels b, d, e and g) or absence (panels a, c and f) of 500 ng/ml anti-human Fas antibody CH11. Cell viability was measured by detecting EGFP-positive cells by fluorescent microscopy. Arrows indicate dead cells. The typical dead cell exhibiting apoptotic bodies was magnified (panel e). (C) Immunocytochemical analysis of transfectants expressing h/oFas. pME18S-h/oFas (panels a and b) or pME18S-hFAS (panels c and d) were cotransfected transiently with phLBR1TM-EGFP into NIH3T3 cells. After culturing for 48 h, transfectants were incubated for 12 h in the presence (panels b and d) or absence (panels a and c) of CH11. Activated Casp3 in cells expressing EGFP in the nucleus was visualized by staining with anti-cleaved Casp3 and fluorescently-labeled secondary antibodies. After counterstaining with DAPI, cells were photographed by fluorescent microscopy. Arrows indicate transfectants. (D) Cytotoxicity assays of Medaka FADD-expressing mammalian cell lines. The pME18S-Flag/oFADD-EGFP plasmid was transfected into HeLa cells (panels a and b) and wild-type (panel c) or *Casp8*-deficent (panel d) MEF cells. Half of the HeLa transfectants were cultured in the presence of 100 μM zVAD-fmk (panel b). After 24 h of culture, cells were washed, fixed, and examined by fluorescence microscopy. Viable cells were defined as EGFP-positive cells, while typical dead cells are shown by arrows. Abbreviations: WT, wild-type; Casp8-KO, *Casp8*-deficient. (E) Cytotoxicity assays of Medaka Casp8-expressing HeLa cells. The pCMV-EGFP/oCasp8 construct, encoding EGFP/oCasp8, was transfected into HeLa cells alone (panels a, b and d) or in conjunction with pCX-CrmA that encoded CrmA (panel c). Half of transfectants expressing EGFP/oCasp8 alone were incubated with 100 μM zVAD-fmk (panels b and d). After 24 h of culture, transfectants were washed, fixed, and examined by fluorescence microscopy. Viable cells were defined as EGFP-positive cells. Surviving cells expressing EGFP/oCaspa8 were examined by confocal laser scanning microscopy (panel d). In panel d, a dotted line demarks the edge of a single cell. (F) The DNA content of transfectants expressing Medaka FADD or caspase-8 was assessed by flow cytometry. Twenty-four hours after transfection, the DNA content of cells transfected with pME18S (panel a), pME18S-Flag/oFADD-EGFP (panels b and c), pCMV-EGFP/oCasp8 (panels d and e) together with pCX-p35 (panels c and e) was analyzed by staining with PI. The percentage indicates the cellular population with sub-G_1 _DNA content.

As the predicted amino acid sequences of Medaka Fas, FADD and Casp8 exhibit homology to their mammalian counterparts, these proteins likely play an equally critical role in the regulation of apoptosis. To confirm the universal function of Fas, FADD and Casp8 in vertebrates, we investigated the killing activity of these Medaka molecules following exogenous expression in mammalian cells. We generated several expression constructs coexpressing enhanced green fluorescent protein (EGFP) (Figure 5A) and transiently introduced them into mammalian cell lines. The cell viability of transfectants expressing Medaka molecules was examined by monitoring EGFP expression by microscopy. The pME18S-h/oFas and pME18S-hFAS plasmids, carrying the chimeric *h/oFas *gene and human *FAS *cDNA, respectively, or the control pME18S empty vector were cotransfected with a construct carrying the *egfp *gene into mouse NIH3T3 cells. After 48 h, transfected cells were stimulated with the agonistic anti-human FAS antibody CH11, a potent apoptotic stimulant specific for human FAS-expressing cells [46], for 14 h. The viability of transfectants bearing the control empty vector was unaffected by the presence of CH11, which does not recognize mouse Fas on NIH3T3 cells (Figure 5B, panel b). In contrast, treatment with CH11 induced cell death in transfectants expressing chimeric h/oFas molecules. Both a reduction in the numbers of EGFP-positive cells and an increase in the numbers of apoptotic bodies were observed (Figure 5B, panels d and e), comparable to those changes seen in human FAS-expressing transfectants (Figure 5B, panel g). To confirm that the cell death induced by chimeric h/oFas is apoptotic cell death, we examined caspase-3 (Casp3) activation, which occurs during apoptosis, in transfectants. The pME18S-h/oFas or pME18S-hFAS plasmid was cotransfected with a construct carrying the *lbr-egfp *fusion gene into NIH3T3 cells. After 48 h, transfected cells were stimulated with or without CH11 for 12 h. Activation of Casp3 in cells expressing EGFP in the nucleus was analyzed by immunocytochemical staining with an anti-cleaved Casp3 antibody. Following proteolytic processing of the inactive zymogen, Casp3 forms subunits and becomes active. Following treatment with CH11, we detected activated Casp3 in EGFP-positive cells coexpressing either h/oFas or hFAS (Figure 5C). However, no Casp3 activation was observed in EGFP-negative or untreated cells (Figure 5C). These data indicate that chimeric h/oFas is a functional molecule with killing activity; the putative death domain in Medaka Fas cytoplasmic tail is capable of transmitting apoptotic signals to mammalian cells in response to exogenous stimuli.

To test the pro-apoptotic activity of Medaka FADD, we transfected pME18S-Flag/oFADD-EGFP into two mammalian cell lines, human HeLa cells and mouse embryonic fibroblasts (MEF) cells, in the presence or absence of the pan-caspase inhibitor, carbobenzoyl-Val-Ala-Asp-fluoromethylketone (zVAD-fmk) (Figure 5D). As HeLa cells preserve the Fas-mediated apoptotic signaling pathway, they are suitable for examining the potency of pro-apoptotic molecules such as FADD and caspase-8. Exogenous expression of Medaka FADD induced cell death in HeLa transfectants, resulting in reductions in the numbers of EGFP-positive cells associated with increased apoptotic bodies (Figure 5D, panel a). The addition of zVAD-fmk, however, inhibited the killing activity of Medaka FADD, permitting the survival of the majority of EGFP-positive cells (Figure 5D, panel b). Using MEF cells isolated from both wild-type and *Casp8*-deficient embryos [47], we examined the pro-apoptotic activity of Medaka FADD. We assumed that *Casp8*-deficient MEF cells, but not wild-type MEF cells, are unable to transmit apoptotic signals triggered by Medaka FADD. Indeed, ectopically expressed Medaka FADD induced cell death in wild-type MEFs, as evidenced by the reduced numbers of EGFP-positive cells and the appearance of apoptotic bodies (Figure 5D, panel c). In contrast, Medaka FADD failed to induce cell death in *Casp8*-deficient MEF cells, as the majority of EGFP-positive cells survived and retained normal morphology (Figure 5D, panel d). To confirm the pro-apoptotic activity of Medaka FADD, we examined the DNA content of cells expressing Medaka FADD by flow cytometry (Figure 5F). Overexpression of Medaka FADD in HeLa cells increased the cellular population in a sub-G_1 _fraction (Figure 5F, 16.0% in panel b), one indicator of apoptosis [48]. In contrast, coexpression of the anti-apoptotic molecule p35, a known pan-caspase inhibitor [49], with Medaka FADD reduced this population of cells (Figure 5F, 3.6% in panel c). These data indicate that Medaka FADD is able to transmit apoptotic signals into transfected cells via activation of mammalian caspase-8.

To assess the pro-apoptotic activity of Medaka caspase-8, the EGFP/oCasp8 fusion protein was expressed in HeLa cells in the absence or presence of either zVAD-fmk or cytokine response modifier A (CrmA) (Figure 5E). Exogenous expression of Medaka Casp8 induced cell death in transfected cells. However, Casp8-induced killing was inhibited by both zVAD-fmk and CrmA. Following transfection, the number of EGFP-positive cells was reduced (Figure 5E, panel a), while the number of EGFP-positive cells increased after culture in zVAD-fmk or following CrmA coexpression (Figure 5E, panels b and c). As CrmA acts as a specific inhibitor by blocking the protease activity of mammalian caspase-8 [50], our data indicate that protease activity is absolutely required for Medaka caspase-8-mediated cell death. Aggregates of death-effector filaments, identified in a previous report [51], were observed in the surviving cells that expressed EGFP/oCasp8 in the presence of either zVAD-fmk or CrmA (Figure 5E, panel d and data not shown). As shown in Figure 5F, expression of Medaka caspase-8 led to an increase in the sub-G_1 _fraction in transfected HeLa cells, but this was abrogated by the co-expression of p35 (12.8% and 3.5% in panels d and e). These data suggest that Medaka caspase-8 is a pro-apoptotic molecule requiring homotypic oligomerization, a characteristic shared by mammalian DED-containing molecules.

## Discussion

In this study, we identified and characterized the DISC components Fas, FADD and caspase-8 from Medaka fish. Our determination of the genomic structure of the *fas*, *fadd *and *caspase-8 *genes revealed that the genomic organization of these genes is preserved between fish and mammals. These gene products also exhibited similar protein structures and functions to their mammalian counterparts. In cytotoxic assays, Medaka Fas, FADD and caspase-8 exhibited pro-apoptotic activity even in mammalian cells. Thus, these results clearly showed that the apoptotic machinery downstream of cell surface death receptors is functional in fish as well as mammals.

**Figure 6:**
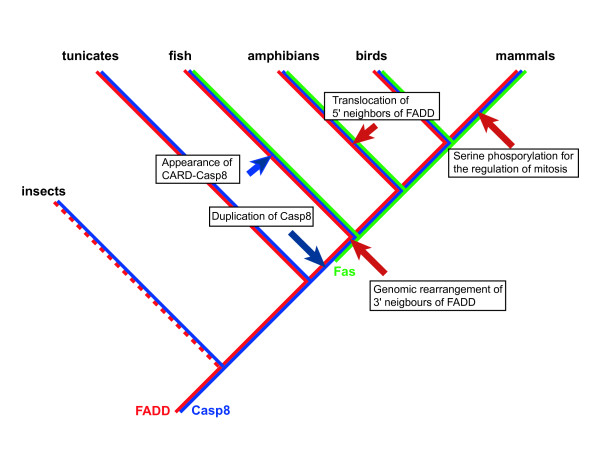
**Summary of evolutionary insights provided in this study**. The constructed molecular phylogenetic trees for Fas, FADD and Casp8 colored with green, red and blue lines, respectively, are shown together. Gene rearrangements and the appearance of divergent genes, domains, or domain architectures are indicated by an arrow at the appropriate evolutionary timepoint. Death receptors including Fas appear to be unique to vertebrates because no orthologs of death receptors have been identified in the decoded genomes of non-vertebrates [41,42,53,54]. Gene rearrangements of the segment including the *fadd *gene were detected by comparative gene mapping in the present study. In addition, the extended C-terminal portion of FADD, which is phosphorylated for the regulation of mitosis [43,44], is uniquely detected in mammals. Early during vertebrate evolution, the *casp8 *and related genes arose from tandem duplication events of an ancestral *casp8 *gene as described [34]. Based on our computational analysis, it was hypothesized that the *card-casp8 *gene was originally generated in the fish lineage by local duplication.

Six death receptors have been identified in mammals [52], and it was assumed that at least one fish ortholog of mammalian death receptors could be found. Indeed, two death receptors, ZH-DR and OTR have been previously identified in zebrafish [20,21]. A recent report proposed that they are counterparts of mammalian TRAIL receptors, but not Fas [28]. In this study, we identified a molecule exhibiting the closest homology to mammalian Fas. We concluded that this molecule is a bone fide fish ortholog for mammalian Fas by several lines of evidence: its domain structure (Figure 1), similarity of its genomic structure (Figure 2), the conservation of synteny of the *Fas *gene-containing genomic region between humans and Medaka (Figure 3), a molecular phylogenetical evidence (Figure 4) and the functionality of the Medaka Fas death domain for the transmission of apoptotic signals (Figure 5). Thus, fish possess several death receptors including Fas that show similar protein structures and potencies to mammalian receptors. In contrast to the fish, no death receptors containing a DD motif have yet been identified in the insect, ascidian and sea urchin genomes [41,42,53,54]. Therefore, we argue that death receptors, such as Fas, appear to be unique to vertebrates (Figure 6). Additionally, further analysis in the genome of jawless vertebrates such as lamprey will be helpful for confirming our inference.

Members of the FADD protein family have been identified and characterized in mammals, amphibians and insects. Although *Drosophila *FADD (dFADD, also termed CG12297-PA) [45] physically interacts with and activates DREDD, a *Drosophila *counterpart of caspase-8, it does not interact with mammalian death receptors or caspase-8 in mammalian cells [45]. There are no orthologs of the death receptor in *Drosophila *[41]. It was recently reported that dFADD acts in concert with DREDD in immune defenses against bacterial infections [55,56], indicating a functional difference in FADD molecules between these species. We determined that Medaka FADD is able to induce apoptosis of mammalian cells. In addition, mammalian caspase-8 is required for the pro-apoptotic activity of Medaka FADD (Figure 5), which demonstrates that FADD function is conserved between fish and mammals. We previously demonstrated that *Xenopus *FADD could substitute functionally for mammalian FADD [36]. There is an evolutional divergence in the function of these proteins between vertebrates and insects. We identified *fadd*-like genes in the ascidian *Molgula tectiformis*, sea urchin *Strongylocentrotus purpuratus *and cnidaria *Hydra magnipapillata *in the GenBank database ([see Additional file 7] and data not shown). The predicted amino acid sequences of these gene products reveal conservation of the DED and DD, but not the C-terminal extension present in mammals. This might be a universal function of FADD that has been conserved from simple Metazoans to vertebrates during evolution. We hypothesize that the adaptor molecule FADD has two functions; one is required for the extrinsic apoptotic signaling pathway in vertebrates, while the second has not yet been defined, but is broadly conserved in bilaterian.

In humans, the *CASP8*, *caspase-10 *(*CASP10*) and *CFLAR *(also termed c-FLIP) genes, which localize in a cluster at the same chromosomal region [57], are thought to be arisen by duplication of an ancestral gene because of their common unique protein structure consisting of two DEDs and a protease domain and high similarity in amino acid sequences. In both birds and amphibians, these *casp8*, *casp10 *and *cflar *genes, which map to the same chromosome, have been identified [34]. We have searched for *casp10 *and *cflar *genes in fish, identifying *casp10*-like and *cflar *genes in the genomic database of zebrafish, Tetraodon and fugu. In these fish, the *casp10 *and *cflar *genes are uniquely segregated to distinct chromosomes [34]. In Medaka, the *casp10-like *and *cflar-like *genes were also found in the genome database, but they localized in the contigs distinct from the *casp8 *gene (data not shown), suggesting the chromosomal segregation of these three genes in Medaka. Interestingly, we identified a gene, which encodes a protease domain highly similar to that of Casp8, next to the *casp8 *gene in the Medaka chromosome (Figure 3C). Based on the full-length sequence data for the *casp8-like *gene, we concluded that this gene represents a distinct caspase type containing the CARD motif from caspase-8 and caspase-10 (Figure 3D) and termed as *card-casp8*. Moreover, this gene product exhibited pro-apoptotic activity similar to Casp8 [see Additional files 5, 8]. Although the present study does not fully address the function of CARD-Casp8, our data suggest that the *casp8 *and *card-casp8 *genes diverged from an ancestral gene by local duplication (Figures 3, 6 and [34]). The mechanisms by which caspase-8 and caspase-10 act independently or compensate for each other in these organisms and by which cflar regulates the activation of these caspases are important to understand the biological significance of these molecules in vertebrates. Further functional analyses of caspase-8 and its related molecules including CARD-Casp8 in fish will help resolve the relationship of caspase function to the divergence observed throughout evolution.

In the catfish, *fas*-, *fadd*- and *casp8-like *genes have been identified [35,39]. As the amino acid sequences predicted from the catfish *fas*-like and *fadd*-like genes exhibited 37% and 43% identities to the Medaka counterparts, these genes are probably also orthologs of mammalian Fas and FADD. The functions of these catfish molecules, however, have not yet been defined. The catfish *casp8*-like gene is not likely to be an ortholog of mammalian *caspase-8 *because this gene product has no DED but possesses a CARD (Figure 3D). Regardless, our study strongly suggests the evolutionary conservation of the pro-apoptotic ability of Fas, FADD and caspase-8, the core components of the extrinsic apoptotic pathway, in Medaka fish. Our data is further supported by a recent report indicating that zebrafish components required for extrinsic pathway induce cell death in embryos when overexpressed [28].

In addition to the *fas*, *fadd *and *caspase-8 *genes, the *caspase-3 *and *bid *genes, whose products play roles as effector and amplifier, respectively, downstream of caspase-8, were identified in the database ([GenBank: BAC00948], [GenBank: BAC00949], and [GenBank: BJ707272]). The Medaka *fas ligand-like *gene was also detected in the Ensembl genome database ([Ensembl: UTOLAPRE05100113859]), and its predicted amino acid sequence was highly similar to Fas ligand previously identified in zebrafish and flounder [28,58]. Thus, the molecular machinery required for the extrinsic pathway fully exists in Medaka fish, and Medaka is a representative model vertebrate suitable for studies of gene function and development [59-61]. Medaka will serve as a powerful tool in which to dissect both the basic mechanisms and the complexities of apoptosis induction throughout evolution.

## Conclusion

Our study clearly demonstrated that Medaka Fas, FADD and casapse-8 are functional molecules possessing pro-apoptotic capacity. As shown in Figure 6, Medaka and probably other fishes have completed these core components essential for the extrinsic pathway while chordates and other invertebrates lack one or more components in their genomes. Therefore, it reaches the conclusion that the apoptotic signaling machinery is evolutionarily conserved throughout vertebrates and this novel apoptotic system has been established after the divergence between vertebrates and non-vertebrates.

## Methods

### Cell lines and reagents

Caspase-8-deficient embryonic fibroblasts (MEFs) isolated from caspase-8-deficient mouse [47], mouse NIH3T3 fibroblasts, and human cervical carcinoma HeLa cells were cultured in Dulbecco's Modified Eagle's medium supplemented with 10% fetal calf serum. An anti-human Fas monoclonal antibody, CH-11, was prepared as described [46]. Anti-cleaved caspase-3 and Alexa Fluor 594-conjugated goat anti-rabbit antibodies were purchased from Cell Signaling Technology Inc. (Beverly, MA) and Molecular Probes Inc. (Eugene, OR), respectively. The VECTASHIELD mounting medium with DAPI and the peptide caspase inhibitor, zVAD-fmk were purchased from Vector Laboratories Inc. (Burlingame, CA) and MBL (Nagoya, Japan), respectively. The pEGFP-C1 and pIRES-EGFP plasmids were purchased from BD-Clontech (Palo Alto, CA).

### Computational analyses and DNA sequencing

To search for Medaka homologs of Fas, FADD and caspase-8 in the GenBank database, we utilized the BLAST program [29] using the human counterparts as a query. Medaka EST clones corresponding to human *FAS *(*TNFRSF6*), ([GenBank: AU176749]), human *FADD *([GenBank: AU242372]) and human *caspase-8 *(*CASP8*) ([GenBank: BJ006125] and [GenBank: AV670945]) were identified. The nucleotide sequences of these four clones were confirmed for both strands using DyeDeoxyterminator Cycle sequencing (Applied Biosystems Inc., Foster City, CA) on automated DNA sequencers (PRISM™ 3100, Applied Biosystems Inc. and LI-COR 4000, LI-COR Biosciences, Lincoln, NE). Similarly, we confirmed the nucleotide sequences of Medaka *card-casp8*, *Xenopus tropicalis fas *and *Molgula tectiformis fadd *cDNA clones (MF01SSB040F15, IMAGE:8956885 and mtgd021e23), which were identified by searching the EST databases.

To determine the genomic structure of the Medaka *fas*, *fadd *and *casp8 *genes, we searched for genomic sequences of these three genes in the Medaka genome database [62] created by the National Institute of Genetics and the University of Tokyo. In scaffold28293, scaffold100324 and scaffold1622, the *fas *gene was partially detected, while the *fadd *and *casp8 *genes were identified in scaffold7231 and scaffold169, respectively. By comparison of the genomic and cDNA sequences, we determined the number of exons and the exon-intron boundaries in these three genes. Similarly, we examined the exon-intron boundaries for the human and chicken *FAS *genes, the human, mouse, chicken, West African clawed frog and two species of fish, stickleback and zebrafish *FADD *genes and the human, stickleback and zebrafish *CASP8 *genes by searching the GenBank (genome sequences for human, chimpanzee, mouse, rat and cow) and Ensembl [63], (contig9.25, contig98.102 and contig75.50 for chicken *fas*, *fadd *and *casp8*, scaffold82 for frog *fadd*, and contig459 and contig8586 for stickleback *fadd *and *casp8*) databases. We also included the fugu genomic sequence (scaffold263 for *casp8*), taken from the Ensembl database, in our comparative mapping. The fish *npsn *(nephrosin) and *mtch2 *(mitochondrial carrier homolog 2) genes and the human *CCND1 *(cyclin D1), *CTTN *(cortactin), *IFIT2 *(interferon-induced protein with tetratricopeptide repeats 2), *MCM6 *(minichromosome maintenance protein 6), *PAPSS2 *(3'-phosphoadenosine 5'-phosphosulfate synthethase 2), *PPFIA1 *{protein tyrosine phosphatase, receptor type, f polypeptide (PTPRF), interacting protein (liprin), α1}, *PTEN *(phosphatase and tensin homolog deleted on chromosome 10) and *TMEM16A *(transmembrane protein 16A) genes and their non-mammalian orthologs, listed in Supplementary Table S4 [see Additional file 4], were used to generate the physical maps.

### Polymerase chain reaction (PCR) analysis

To determine the splice acceptor and donor sequences adjacent to exon 2 of the *fas *gene, we isolated genomic DNA from adult fish and amplified the desired sequence by PCR using following primer sets; to amplify the 1^st ^intron, we utilized a forward primer (5'-AGTGAAGTTGATCATGGCGAC-3') from the 1^st ^exon and a reverse primer (5'-CACGTTTTACCGTCGTGTGTGTAG-3') from the 2^nd ^exon, while to detect the 2^nd ^intron, we used a forward primer (5'-TGGAAGCTACACACACGACGGTAA-3') from the 2^nd ^exon and a reverse primer (5'-TTCTTCGCAGAAGCCACATTCACC-3') from the 3^rd ^exon. Following an initial denaturation at 94°C for 5 min, we performed 30 cycles of PCR at 96°C for 15 sec, 55°C for 15 sec and 72°C for 8 min in the presence of LA Taq DNA polymerase (TAKARA Bio Inc., Otsu, Japan). Amplified PCR products were subcloned into thepCRII plasmid (Invitrogen, Carlsbad, CA) and sequenced.

### Molecular phylogenetic analyses

The amino acid sequences for the Fas proteins from catfish, chicken, human, mouse and zebrafish published in the GenBank database were included for analysis as listed in Supplementary Table S5 [see Additional file 7]. Similarly, we selected the amino acid sequences of the FADD proteins from catfish, chicken, human, mouse and zebrafish [see Additional file 7]. In addition, we predicted the amino acid sequence for FADD from West African clawed frog and stickleback in the Ensembl genome database [see Additional file 7]. To confirm correct tree construction, we also cited the human, chicken and zebrafish TRADD (tumor necrosis factor receptor 1-associated death domain protein) sequences including a death domain and compared the amino acid sequences of Fas and FADD in our analysis. We aligned the death domains of these proteins using T-COFFEE program [64] and manually removed gaps to construct the phylogenetic tree. Based on this alignment, a molecular phylogenetic tree was constructed using the neighbor-joining method implemented in the MEGA program [65,66].

### Construction and transfection of expression vectors

To express Medaka FADD and caspase-8 in mammalian cell lines, we generated two mammalian expression plasmids encoding these proteins. The pME18S-Flag/oFADD-EGFP plasmid was generated by cloning the Medaka *fadd *cDNA, amplified by PCR, into the modified pME18S plasmid [67] containing a Flag-tag in the 5' region and an internal ribosome entry site (IRES)-EGFP in the 3' region. The pCMV-EGFP/oCasp8 plasmid was created by inserting the Medaka *caspase-8 *cDNA, amplified by PCR, into pEGFP-C1. To test the killing activity of Medaka Fas, we generated a chimeric gene *h/oFas *by PCR amplification. The DNA fragment encoding the extracellular region of human Fas was ligated to the DNA fragment encoding the transmembrane and cytoplasmic regions of Medaka Fas. This fragment was cloned into pME18S to generate pME18S-h/oFas. The viral gene encoding CrmA, the kind gift of Dr. D. J. Pickup (Duke University), was cloned into the pCAGGS expression vector [68] to generate pCX-CrmA. The pCX-p35 plasmid was generated by cloning the baculovirus *p35 *gene into the mammalian expression vector pCAGGS as described previously [36]. The phLBR1TM-EGFP plasmid, which encodes a nuclear fusion protein consisting of LBR and EGFP (a kind gift of Dr. J. Ellenberg, EMBL), was used for both immunocytochemical and flow cytometric analyses.

Transfection of these plasmid constructs into cells was performed using LipofectAMINE PLUS Reagent (Invitrogen), according to manufacturer's instructions.

### Cytotoxicity assays

After transfection with pME18S-Flag/oFADD-EGFP or pCMV-EGFP/oCasp8, HeLa and *Casp8*-deficient MEF cells were incubated for 24 h in the presence or absence of 100 μM zVAD-fmk or with or without viral protein CrmA. Following fixation in PBS containing 3.7% formaldehyde, we examined the number of EGFP-positive cells by fluorescence microscopy (DMIRE2, Leica Microsystems, Wetzlar, Germany). Magnified fluorescent images of transfectants were obtained on a confocal laser scanning microscope (TCS SP2, Leica Microsystems). We transfected the pME18S-h/oFas and pME18S-hFAS plasmids, which encode chimeric h/oFas or human FAS, respectively, with pEGFP-C1 into NIH3T3 cells. Forty-eight hours post-transfection, we examined the killing activity of h/oFas and human FAS following treatment for 14 h with 500 ng/ml of anti-human Fas antibody CH11.

### Immunocytochemical analysis

To confirm that the h/oFas-mediated death signal activates the apoptotic signaling pathway, we examined caspase-3 activation in transfected cells using an immunocytochemical method. Briefly, 48 h post-transfection with pME18S-h/oFas or pME18S-hFAS together with phLBR1TM-EGFP, which was used for detection of transfected cells, NIH3T3 cells were treated for 12 h without or with 500 ng/ml of CH11 and fixed in PBS containing 4% paraformaldehide for 10 min. After washing once with PBS, cells were permeabilized in 0.2% Triton X-100/PBS for 5 min, washed three times for 5 min each with PBS and quenched in PBS containing 0.1% sodium borohydride for 5 min. After washing once with PBS, cells were immersed in blocking buffer {PBS containing 10% goat serum and 1% bovine serum albumin (BSA)} for 1 h. Cells were washed once with PBS and incubated with an anti-cleaved caspase-3 antibody in 1% BSA in PBS overnight at 4°C. After washing three times for 5 min each with PBS, cells were incubated with the fluorescently-labeled secondary antibody (Alexa Fluor 594 goat anti-rabbit antibody) in 1% BSA in PBS for 1 h, washed three times for 5 min each with PBS and mounted with a coverslip using VECTASHIELD mounting medium. Caspase-3 activation in cells expressing EGFP in the nucleus was visualized by fluorescence microscopy (Axioplan, Carl Zeiss GmbH, Jena, Germany).

### Flow-cytometric analysis

Twenty-four hours after transfection with pME18S-Flag/oFADD-EGFP or pCMV-EGFP/oCasp8 with or without pCX-p35, HeLa cells were fixed in 70% ethanol at -20°C for 1 h as described previously [36]. For selection of transfected and untransfected cells, we co-transfected cells with phLBR1TM-EGFP. Following fixation, cells were washed with PBS, treated with RNase A (50 μg/ml) in PBS at 37°C for 30 min and stained with 50 μg/ml of propidium iodide (PI) in PBS for 30 min. The DNA content of cells expressing EGFP in the nuclei was then analyzed by flow cytometry (XL™, BECKMAN-COULTER, Miami, FL).

## List of abbreviations used

BSA, bovine serum albumin; CARD, caspase recruitment domain; ccnd1, cyclin D1; CrmA, cytokine response modifier A; CTTN, cortactin; DD, death domain; DED, death effector domain; DISC, death-inducing signaling complex; EGFP, enhanced green fluorescent protein; EST, expressed sequence tag; FADD, Fas-associated death domain protein; IFIT2, interferon-induced protein with tetratricopeptide repeats 2; IRES, internal ribosome entry site; MCM6, minichromosome maintenance protein 6; MEF, mouse embryonic fibroblasts; mtch2, mitochondrial carrier homolog 2; npsn, nephrosin; OTR, ovarian tumor necrosis factor receptor; PAPSS2, 3'-phosphoadenosine 5'-phosphosulfate synthethase 2; PCR, polymerase chain reaction; PI, propidium iodide; PPFIA1, protein tyrosine phosphatase, receptor type, f polypeptide (PTPRF), interacting protein (liprin), α1; PTEN, phosphatase and tensin homolog deleted on chromosome 10; TMEM16A, transmembrane protein 16A; TRADD, tumor necrosis factor receptor 1-associated death domain protein; TRAIL, tumor necrosis factor-related apoptosis-inducing ligand; ZH-DR, zebrafish hematopoietic death receptor; zVAD-fmk, carbobenzoyl-Val-Ala-Asp-fluoromethylketone.

## Authors' contributions

KS performed the cytological and computational analyses, processed all the data and wrote the manuscript. MN was responsible for sequencing of Medaka and *Xenopus *cDNA clones. KK carried out the molecular analysis of the Medaka *fas *gene. YS achieved the phylogenetic analysis and helped to draft the manuscript. All authors read and approved the final manuscript.

## Supplementary Material

Additional file 1 Exon/intron boundaries of the Medaka *fas *gene. The nucleotide sequences of the exon-intron boundaries in the Medaka *fas *gene were indicated as Table S1. Click here for file

Additional file 2 Exon/intron boundaries of the Medaka *fadd *gene. The nucleotide sequences of the exon-intron boundaries in the Medaka *fadd *gene were indicated as Table S2. Click here for file

Additional file 3 Exon/intron boundaries of the medaka *caspase-8 *gene. The nucleotide sequences of the exon-intron boundaries in the Medaka *caspase-8 *gene were indicated as Table S3. Click here for file

Additional file 4 List of animals and gene ID numbers. For the generation of the physical map, animals and gene ID numbers were listed in Table S4. Click here for file

Additional file 5 Supplementary information. The descriptions on the structure of the newly identified *Xenopus fas *gene and the function of the Medaka CARD-Casp8 molecule. The supplemental explanation for Figure S1 and S2 was also reported. Click here for file

Additional file 6 Primary structure of *Xenopus *Fas. The data provided as Figure S1 represent a physical map of the region containing the *Xenopus fas *gene and the alignment of *Xenopus *and human Fas proteins. Click here for file

Additional file 7 List of animals and protein ID numbers. For the generation of a molecular phylogenetic tree, animals and protein ID numbers were listed in Table S5. Click here for file

Additional file 8 Medaka CARD-Casp8 possessing pro-apoptotic activity. Cytotoxicity assay of Medaka CARD-Casp8-expressing HeLa cells was examined and its result was presented as Figure S2. Click here for file
