# Supplementary material for: The evolutionary conservation of the core components necessary for the extrinsic apoptotic signaling pathway, in Medaka fish
Source: BMC Genomics. 2007 Jun 1;8:141. doi: 10.1186/1471-2164-8-141 (PMC1903365; doi:10.1186/1471-2164-8-141)
Supplement: Additional file 1 — Exon/intron boundaries of the Medaka fas gene. The nucleotide sequences of the exon-intron boundaries in the Medaka fas gene were indicated as Table S1. [file 1471-2164-8-141-S1.pdf]

Table S1. Exon/intron boundaries of the Medaka *fas* gene.

Exon	*Splicing donor*	(Intron)	*Splicing acceptor*	Exon
TAGGAACATT<br>leGlyThrLe<br>20	***gt**aagttaat*----	(Intron 1)	----*tgtcgttc**ag***	GGTTCCTAGT<br>eValProSer<br>22
TGTGCTCCTG<br>CysAlaProG<br>50	***gt**aagtctct*----	(Intron 2)	----*catgttct**ag***	GTCAGAAAGT<br>lyGlnLysVa<br>52
CACCCCAACG<br>HisProAsnA<br>96	***gt**ataaatcc*----	(Intron 3)	----*atcttttc**ag***	CCAATTTGGA<br>laAsnLeuGl<br>98
CCTGTGATAT<br>erCysAspIl<br>135	***gt**aagtaagc*----	(Intron 4)	----*cccctata**ag***	ATGTGAATCC<br>eCysGluSer<br>137
GAAAGCCAAG<br>GluSerGlnG<br>158	***gt**aaggaatt*----	(Intron 5)	----*ttttaatc**ag***	AGCCAAGCGA<br>lyIleSerAl<br>160
AAAACAAGAC<br>LysThrArgG<br>189	***gt**atgtcgtt*----	(Intron 6)	----*tttaaatc**ag***	AGCTGAGCGA<br>lnLeuserAs<br>191
AGACAGCGAG<br>oAspSerGlu<br>200	***gt**gagttcat*----	(Intron 7)	----*tgcctcac**ag***	GAACTTGAGT<br>GluLeuGluT<br>201
TATCTCAACG<br>TyrLeuAsnG<br>206	***gt**aagaaaaa*----	(Intron 8)	----*caaatccc**ag***	AAGTGGACAT<br>luValAspIl<br>208

Exon sequence is shown by capital letters; intron sequence by lower case.
Numbers indicate amino acid residue position.
