# Supplementary material for: The evolutionary conservation of the core components necessary for the extrinsic apoptotic signaling pathway, in Medaka fish
Source: BMC Genomics. 2007 Jun 1;8:141. doi: 10.1186/1471-2164-8-141 (PMC1903365; doi:10.1186/1471-2164-8-141)
Supplement: Additional file 2 — Exon/intron boundaries of the Medaka fadd gene. The nucleotide sequences of the exon-intron boundaries in the Medaka fadd gene were indicated as Table S2. [file 1471-2164-8-141-S2.pdf]

Table S2 Exon/intron boundaries of the Medaka *fadd* gene.

Exon	*Splicing donor* (Intron) *Splicing acceptor*	Exon
GAGGAGACAG<br>GluGluThrA<br>101	***gt****accgaacc*---- (Intron 1) ----*gctccctc****ag***	CCAAGCTGAA<br>spLysLeuAs<br>103

Exon sequence is shown by capital letters; intron sequence by lower case.
Numbers indicate amino acid residue position.
