# Supplementary material for: The evolutionary conservation of the core components necessary for the extrinsic apoptotic signaling pathway, in Medaka fish
Source: BMC Genomics. 2007 Jun 1;8:141. doi: 10.1186/1471-2164-8-141 (PMC1903365; doi:10.1186/1471-2164-8-141)
Supplement: Additional file 3 — Exon/intron boundaries of the medaka caspase-8 gene. The nucleotide sequences of the exon-intron boundaries in the Medaka caspase-8 gene were indicated as Table S3. [file 1471-2164-8-141-S3.pdf]

Table S3. Exon/intron boundaries of the medaka *caspase-8* gene.

Exon	*Splicing donor*	(Intron)	*Splicing acceptor*	Exon
GTGGACATTG	***gt***_aagtcacg_----	(Intron 1)	----_gctttgac_***ag***	CACTAAAAGG
TTTGGAGGGG<br>gLeuGluGly<br>36	***gt***_aagaactg_----	(Intron 2)	----_tttcatgc_***ag***	ATCAGTTCAG<br>IleSerSerA<br>37
CTGATTACAG<br>erAspTyrAr<br>93	***gt***_aaaagcag_----	(Intron 3)	----_tctccatc_***ag***	AGTGATGCTG<br>gValMetLeu<br>95
GAAGAGCAAA<br>uLysSerLys<br>129	***gt***_gagcaaat_----	(Intron 4)	----_ctaattac_***ag***	ACTGCTCTGG<br>ThrAlaLeuA<br>130
TACATGCAAG<br>TyrMetGlnA<br>172	***gt***_attagatt_----	(Intron 5)	----_ctttttcc_***ag***	ATCATCGTGG<br>spHisArgGl<br>174
CTATCAAAAG<br>sTyrGlnLys<br>186	***gt***_gtgtttgt_----	(Intron 6)	----_tttcctgc_***ag***	AGACCCAGCA<br>ArgProSerI<br>187
GATCCAAACA<br>AspProAsnA<br>211	***gt***_aaacatca_----	(Intron 7)	----_aatcaaac_***ag***	ATGGAGTAAG<br>snGlyValAr<br>213
ACCTGATGAG<br>rProAspGlu<br>230	***gt***_aacattct_----	(Intron 8)	----_tgccctgt_***ag***	GAGGAGTATT<br>GluGluTyrT<br>231
AAAGATGAAA<br>LysAspGluL<br>268	***gt***_aagtttgt_----	(Intron 9)	----_ttttccac_***ag***	AGTCTCTCAA<br>ysSerLeuAs<br>270
TGATGCTTTG<br>pAspAlaLeu<br>310	***gt***_gagtcatg_----	(Intron 10)	---_tgtcctat_***ag***	GTAGTCTGTG<br>ValValCysV<br>312
CTGCAGAGAG<br>laAlaGluSe<br>435	***gt***_gagaaaca_----	(Intron 11)	---_ccttttc_***ag***	CTCAAAGATG<br>rSerLysMet<br>437

Exon sequence is shown by capital letters; intron sequence by lower case.
Numbers indicate amino acid residue position.
