# Supplementary material for: The evolutionary conservation of the core components necessary for the extrinsic apoptotic signaling pathway, in Medaka fish
Source: BMC Genomics. 2007 Jun 1;8:141. doi: 10.1186/1471-2164-8-141 (PMC1903365; doi:10.1186/1471-2164-8-141)
Supplement: Additional file 4 — List of animals and gene ID numbers. For the generation of the physical map, animals and gene ID numbers were listed in Table S4. [file 1471-2164-8-141-S4.pdf]

Table S4. List of animals and their sequence data cited for the generation of the physical map.

Gene	Identification number
A. Human (*Homo sapiens*)	
CTTN	NM_005231
FADD	NM_003824
FAS	NM_000043
IFIT2	M14659/M14660
PAPSS2	NM_004670
PPFIA1	NM_177423
PTEN	NM_000314
TMEM16A	NM_018043
B. Chicken (*Gallus gallus*)	
cttn	NM_205468
fadd	XM_421073
fas	XM_421659
ifit2	XM_421662
papss2	XM_421558
ppfia1	XM_421074
pten	XM_421555
tmem16a	XM_421071
C. Frog (*Xenopus tropucalis*)	
ccnd1	AAH74566
cttn	NM_00101314
fas	EF555573
fadd	ENSXETG00000003799*
papss2	ENSXETG00000009264*
ppfia1	ENSXETG00000003798*
pten	ENSXETG00000009268*
D. Medaka (*Oryzias latipes*)	
card-casp8	AM144512/EF564191
fas	AAS91707
mcm6	BJ020331/AM138174
mtch2	AAH55216
npsn	AB256945
papss2	MF01SSB007O11
pten	gene13075[#]
tmem16a	XM_685398
E. Zebrafish (*Danio rerio*)	
fas-like	ENSDARG00000043586*
ifit2	ENSDARG00000043584*
papss2	NM_212562
ptena	NM_001001822
F. Fugu (*Takifugu rubripes*)	
casp8	NEWSINFRUP00000179400*
card-casp8	NEWSINFRUP00000182829*
fas-like	Q90XY4*
papss2	Q90XY2*
pten	Q90XY3*
mcm6	NEWSINFRUP00000137818*
G. Stickleback (*Gasterosteus aculeatus*)	
card-casp8	ENSGACT00000017012*
casp8	ENSGACG00000012842*
fadd	ENSGACG00000014400*
mtch2	ENSGACG00000014423*
npsn	ENSGACG00000014415*
tmem16a	ENSGACG00000014393*

Sequence data indicted by asterisks (*) and sharps ([#]) were published in the Ensembl and UTGB genome databases and the other sequence data were published in the GenBank database. Abbreviated genes: *card-casp8*, caspase-8 homolog with a CARD domain; *ccnd1*, cyclin D1; *CTTN*, cortactin; *FADD*, Fas-associated death domain protein; *FAS*, TNFR superfamily member 6; *IFIT2*, interferon-induced protein with tetratricopeptide repeats 2; *mtch2*, mitochondrial carrier homolog 2; *npsn*, nephrosin; *PAPSS2*, 3'-phosphoadenosine 5'-phosphosulfate synthethase 2; *PPFIA1*, protein tyrosine phosphatase, receptor type, f polypeptide (PTPRF), interacting protein (liprin), α1; *PTEN*, phosphatase and tensin homolog deleted on chromosome 10; *ptena*, phosphatase and tensin homolog A; *TMEM16A*, transmembrane protein 16A.
