# Supplementary material for: The evolutionary conservation of the core components necessary for the extrinsic apoptotic signaling pathway, in Medaka fish
Source: BMC Genomics. 2007 Jun 1;8:141. doi: 10.1186/1471-2164-8-141 (PMC1903365; doi:10.1186/1471-2164-8-141)
Supplement: Additional file 5 — Supplementary information. The descriptions on the structure of the newly identified Xenopus fas gene and the function of the Medaka CARD-Casp8 molecule. The supplemental explanation for Figure S1 and S2 was also reported. [file 1471-2164-8-141-S5.doc]

**SUPPLEMENTARY INFORMATION**

###### **Identification of Xenopus Fas**

As shown in Figure 3A, the genomic organization surrounding the *Fas* gene locus is well conserved among the vertebrate genomes. Although the *fas* gene was not identified in amphibians, comparative gene mapping analysis suggests that the *fas* gene should be located between the *pten* and *ifit2* genes in amphibians. We examined the West African clawed frog (*Xenopus tropicalis*) genome sequence in the Ensembl database and identified a *fas-like* gene that localizes close to the *pten* gene (Supplementary Figure S1A) [see Additional file 6]. Furthermore, we searched the GenBank DNA database for the identification of the cDNA corresponding to the *fas-like* gene and identified a candidate EST clone ([GenBank:EL657295]) in the *X. tropicalis* cDNA library. Sequencing of the EST clone indicated that the full-length cDNA encodes an open reading frame of 320 amino acids with characteristics of a death receptor consisting of three cysteine repeat domains in the extracellular region, a transmembrane region and a DD in the cytoplasmic region (Supplementary Figure S1B) [see Additional file 6]. By comparing the genomic and cDNA sequences, we confirmed that the *Xenopus* *fas-like* gene consists of 11 exons and 10 introns, corresponding to those of the human, chicken and Medaka *FAS* genes with coincident splice junction sites (Supplementary Figure S1B) [see Additional file 6]. Therefore, these results indicated that the identified *fas-like* gene is an amphibian *fas* gene. Alignments of the *Xenopus* and human Fas revealed 35% identity and 48% similarity at the amino acid sequence level. Additionally, comparison of *Xenopus* Fas to the death receptors xDR-M1 and xDR-M2, which were previously identified in African clawed frog (*Xenopus laevis*) [1], showed 26% and 22% identity at the amino acid level, respectively. These data support our conclusion that *Xenopus* Fas is more structurally similar to human FAS than to xDR-M1 and xDR-M2. Thus, we identified an amphibian ortholog of mammalian Fas by the strength of chromosome mapping analysis.

###### **Pro-apoptotic activity of Medaka CARD-Casp8**

To investigate the ability of CARD-Casp8 to act as a caspase, we generated a plasmid construct carrying the *card-casp8* gene for expression in mammalian cell lines. The pCMV-Flag/CARDCasp8 plasmid was generated by inserting the *card-casp8* partial cDNA (clone: MF01SSB040F15), which includes a protease domain, into the pCMV-Tag2C expression vector (Stratagene, La Jolla, CA), and this construct was then transfected into HeLa cells. The pro-apoptotic activity of CARD-Casp8 to HeLa cells was examined in the absence or presence of either zVAD-fmk or cytokine response modifier A (CrmA), and the survival of transfectants expressing CARD-Casp8 was determined by monitoring EGFP-expressing cells by microscopy. As shown in Supplementary Figure S2 [see Additional file 8], the number of EGFP-positive cells was reduced by expression of CARD-Casp8 (panels a and b) while those EGFP-positive cells increased after culture in the presence of zVAD-fmk or following CrmA coexpression (panels c and d). These data indicated that exogenous expression of Medaka CARD-Casp8 induces the death of mammalian cells, and this cytotoxicity is dependent on the protease activity of CARD-Casp8 because caspase inhibitors efficiently suppress cell death. In particular, the pro-apoptotic activity of CARD-Casp8 was inhibited by CrmA, a specific inhibitor of Casp8 [2], suggesting that this molecule is functionally similar to Casp8. Consequently, we concluded that CARD-Casp8 is a functional molecule possessing pro-apoptotic activity

**References**

1. Tamura K, Noyama T, Ishizawa YH, Takamatsu N, Shiba T, Ito, M: **Xenopus death receptor-M1 and -M2, new members of the tumor necrosis factor receptor superfamily, trigger apoptotic signaling by differential mechanisms**. *J Biol Chem* 2004, **279:**7629-7635.

2. Zhou Q, Snipas S, Orth K, Muzio M, Dixit VM, Salvesen GS: **Target protease specificity of the viral serpin CrmA. Analysis of five caspases**. *J Biol Chem* 1997, **272:**7797-7800.

**Supplemental explanation for Figure S1 and S2**

**Figure S1**

(**A**) A physical map of the region containing the *Xenopus fas* gene. In the *Xenopus tropicalis* genome, the *fas* gene is located close to the *pten* and *pappss2* genes. (**B**) Alignment of *Xenopus* and human Fas. Deduced amino acid sequences of *Xenopus* and human Fas proteins were compared using the computer alignment program CLUSTAL W. Identical and similar amino acids between *Xenopus* and human Fas sequences are indicated with red or purple, respectively. Arrowheads on amino acid alignment indicate the splice junction sites. The three lines underneath the sequence indicate the cysteine repeat domains (CRDs) in the extracellular region. The double line and the dotted line indicate the transmembrane region and the death domain (DD) within the cytoplasmic region, respectively.

**Figure S2**

Empty vector (panel a) or pCMV-Flag/CARDCasp8 (panels b and c) were cotransfected transiently with pEGFP-C1 into HeLa cells or in conjunction with pCX-CrmA (panel d). Half of the transfectants expressing CARD-Casp8 were incubated with 100 μM zVAD-fmk (panel c). After 24 h in culture, transfectants were fixed, counterstained with DAPI and photographed by fluorescence microscopy. Viable transfectants were defined as EGFP-positive cells. Assays are representative of three independent experiments.
