# Supplementary material for: The evolutionary conservation of the core components necessary for the extrinsic apoptotic signaling pathway, in Medaka fish
Source: BMC Genomics. 2007 Jun 1;8:141. doi: 10.1186/1471-2164-8-141 (PMC1903365; doi:10.1186/1471-2164-8-141)
Supplement: Additional file 6 — Primary structure of Xenopus Fas. The data provided as Figure S1 represent a physical map of the region containing the Xenopus fas gene and the alignment of Xenopus and human Fas proteins. [file 1471-2164-8-141-S6.pdf]

Additional file 6 (Figure S1)

A

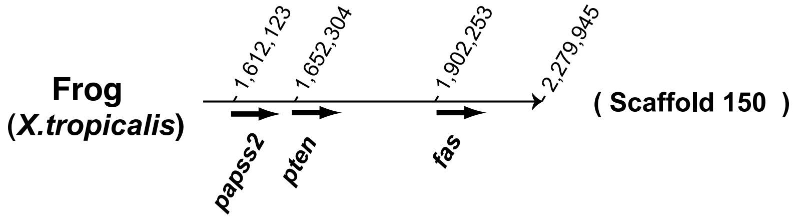

B

```
xFas MLLPWICLFIALAAERNSASAINNIGDTSPAYTGKPSAPLRR-FFKRELKCEDGEYPGNQ  59
hFAS MLGIWTLLPLVLTSVARLSSKSVNAQVTDINSKG---LELRKTVTTVETQNLEGLHHDGQ  57

xFas YCCKNCPAGTYVENDCQENHQKPNCQPCTDGKDYMDKPNGYHQCLLCKRCDPEQGEDVHS 119
hFAS FCHKPCPPGERKARDCTVNGDEPDCVPCQEGKEYTDKAHFSSKCRRCRLCDEGHGLEVEI 117

xFas PCTVFRNTVCKCKVNFFCGTNSTQDPRSCDHCQPCTQCEKGVAESCTETRDTVCNK-GSR 178
hFAS NCTRTQNTKCRCKPNFFC--NST----VCEHCDPCTKCEHGIIKECTLTSNTKCKEEGSR 171

xFas YRWGLLAALILVAVGAGLLCV--------RCRQKPRPIYQPPTLTPLVKPYPSHLEDIDL 230
hFAS SNLGWLCLLLLPIPLIVWVKRKEVQKTCRKHRKENQGSHESPTLNPETVAIN--LSDVDL 229

xFas ETPLQDLADIMLHDDVVKCVRRMGLSNPTIDDIKNTNGQG-REGRYQLLRSWYVQYGQMG 289
hFAS SKYITTIAGVMTLSQVKGFVRKNGVNEAKIDEIKNDNVQDTAEQKVQLLRNWHQLHGKKE 289

xFas ALRHLIKTLRDQGLNKPADDFIDILNRRVQP                              320
hFAS AYDTLIKDLKKANLCTLAEKIQTIILKDITSDSENSNFRNEIQSLV              335
```
