# Supplementary material for: The evolutionary conservation of the core components necessary for the extrinsic apoptotic signaling pathway, in Medaka fish
Source: BMC Genomics. 2007 Jun 1;8:141. doi: 10.1186/1471-2164-8-141 (PMC1903365; doi:10.1186/1471-2164-8-141)
Supplement: Additional file 7 — List of animals and protein ID numbers. For the generation of a molecular phylogenetic tree, animals and protein ID numbers were listed in Table S5. [file 1471-2164-8-141-S7.pdf]

Table S5. List of animals and their sequence data cited for the generation of a phylogenetic tree.

Animals	Genus name	Identification number		
		Fas	FADD	TRADD
Ascidian	*Molgula tectiformis*		CJ394055	
Catfish	*Ictalurus punctatus*	AAT36327	AAS84609	
Chicken	*Gallus gallus*	Q9DGH7	ENSGALP00000012326*	XP_414067
Human	*Homo sapiens*	P25445	Q13158	Q15628
Medaka	*Oryzias latipes*	AAS91707	AAS91703	
Mouse	*Mus musculus*	P25446	Q61160	
Stickleback	*Gasterosteus aculeatus*		ENSGACP00000018996*	
West African clawed frog	*Xenopus tropicalis*	EF555573	ENSXETP00000008273*	
Zebrafish	*Danio rerio*	DQ812117^	BC114285	BC083425

Sequence data indicted by asterisks (*) were published in the Ensembl database and the other sequence data were published in the GenBank database. Sequence data indicted by circumflex (^) was incomplete.
