# Supplementary figures and images for: The evolutionary conservation of the core components necessary for the extrinsic apoptotic signaling pathway, in Medaka fish
Source: BMC Genomics. 2007 Jun 1;8:141. doi: 10.1186/1471-2164-8-141 (PMC1903365; doi:10.1186/1471-2164-8-141)

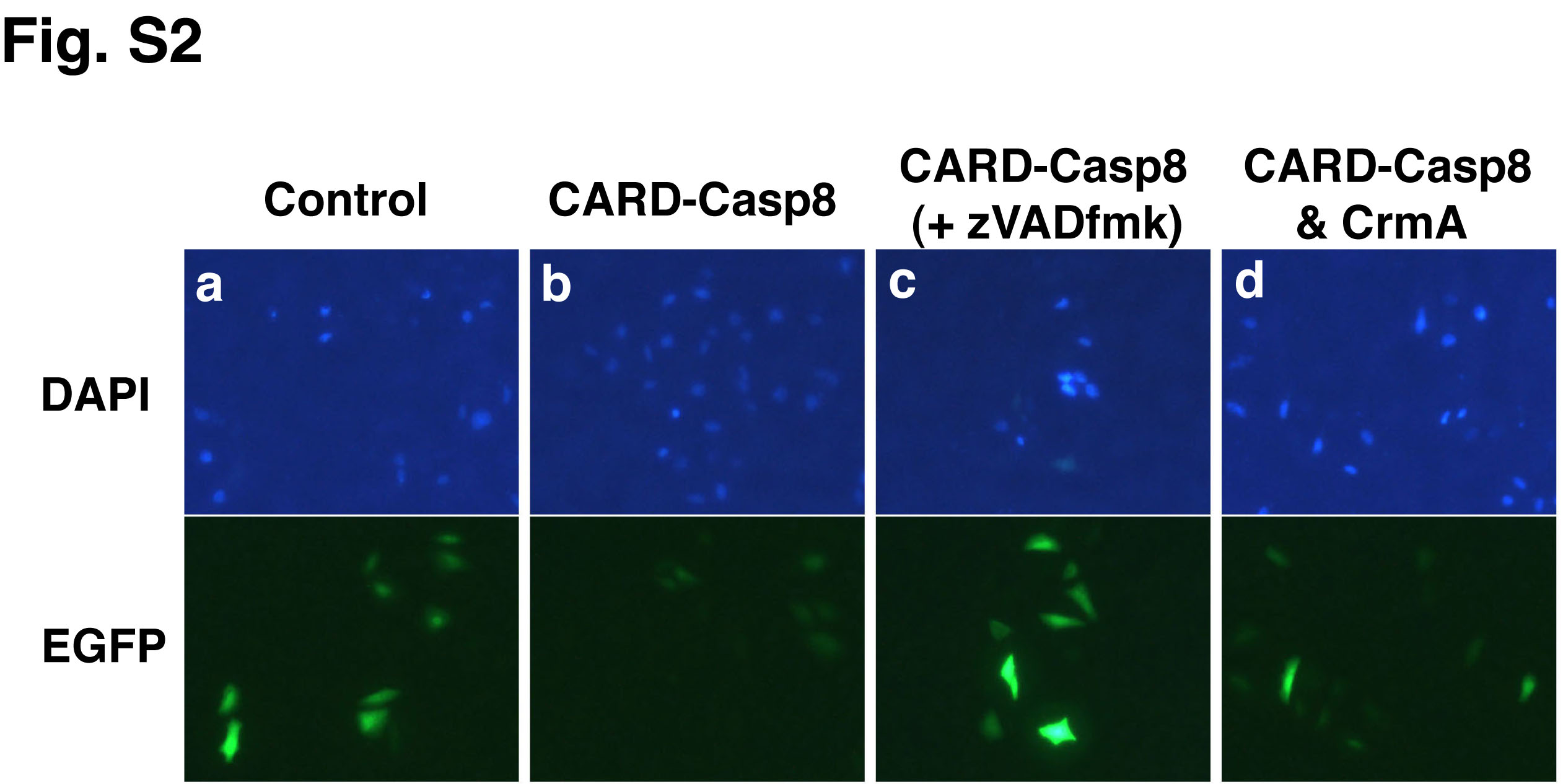

Supplement: Additional file 8 — Medaka CARD-Casp8 possessing pro-apoptotic activity. Cytotoxicity assay of Medaka CARD-Casp8-expressing HeLa cells was examined and its result was presented as Figure S2. [file 1471-2164-8-141-S8.jpeg]
